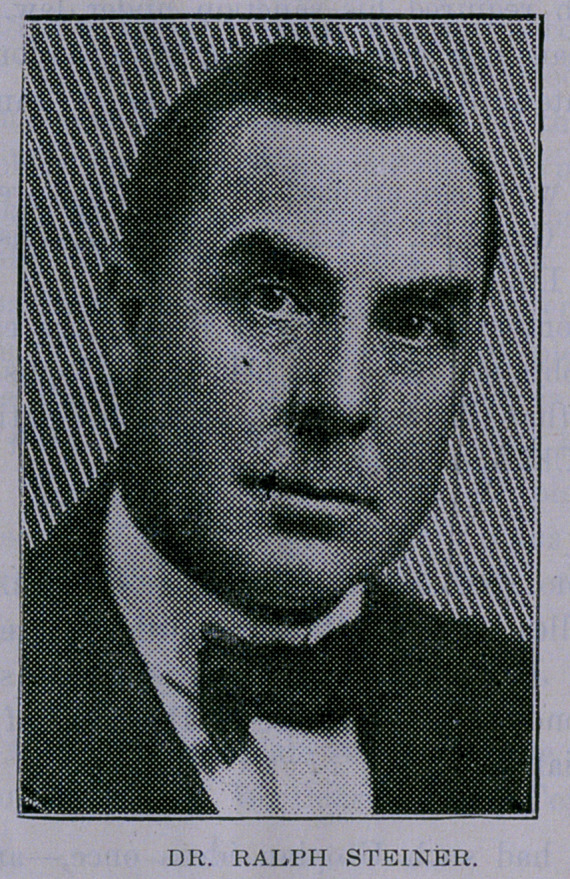# Registrar of Vital Statistics

**Published:** 1911-02

**Authors:** 


REGISTRAR OF VITAL STATISTICS.—John E. Rosser, late Secretary to the University of Texas, and *ad interim* Staff Correspondent of the *Houston Post* and other papers, has been appointed Registrar of Vital Statistics. The appointment is an excellent one. Mr. Rosser is well qualified in every way for this important work. Vital statistics is a distinct science, and no mere scribe can handle it.

DR. RALPH STEINER.

THE APPOINTMENT OF DR. RALPH STEINER, of Austin, to the highest medical position in the State,—State Health Officer and President of the State Board of Health,—was a master-stroke,—an inspiration; for Dr. Steiner, besides possessing every qualification for the great work, is universally beloved, admired and respected by all who know him. To a thorough knowledge of medicine and sanitation, a large experience as a physician, and for many years a specialist, he adds a charming personality, a genial, sunny disposition, and high-bred courtesy that distinguish him and endear him to hosts of attached friends—lay and professional. We have the satisfaction of presenting his likeness and brief sketch of his life to our readers. Dr. Steiner will be supported by six exceptionally strong men, a most excellent combination, towit: Drs. A. W. Fly, B. M. Worsham, B. F. Calhoun, K. M. Beall, H. L. McLaurin and S. M. Lister.

Ralph Steiner, M. D., President Texas State Board of Health, born at Austin, Texas, February 5, 1859; educated in the University of the South, Sewanee, Tennessee; graduated in medicine, University of Maryland, March, 1883.

Dr. Steiner was Consul at Munich four years under the Cleveland administration. During his stay in Europe he perfected himself in his chosen specialty—diseases of the ear, nose, throat and chest. His father was a physician.

Legislative, et cetera.—A bill to prevent the marriage of persons afflicted with tuberculosis, syphilis, gonorrhea, insanity, epilepsy, idiocy, etc.—requiring a physician's certificate as a condition to a marriage license—has been introduced into the Legislature and reported upon favorably by the House Committee on Public Health. It was prepared by Representative Nickels of Hunt, and fathered by the State Society on Social Hygiene. This society met in Austin in a two days' session and carried out the program announced in our January number. Dr. M. Duggan, of San Antonio, President of the Society, had charge of the bill and advocated it before the committee in a strong speech. He was supported by Dr. T. Y. Hull, of San Antonio, the Secretary, and by Rev. Geo. D. Harris, of San Antonio, and by Dr. Jno. T. Moore, President of the Texas State Medical Association, with some suggestions for amendment; and,—in its main object and purpose,—by Dr. Daniel; also by Dr. Johnson, of Giddings, a former Representative.

Strains at a Gate and Swallows a Sawmill.—The Senate would abolish the cigarette, but the holy fetich—the saloon—is sacred. Hands off!

With Six Doctors in the House, the medical profession can reasonably hope to secure much needed sanitary legislation. It is very gratifying to state that Governor Colquitt, in his message, kept his promises made during the campaign, to recommend the Tuberculosis Sanitarium, the Leprosarium, the strengthening of the Public Health Department by the appointment of additional force, etc. It will be remembered that the Thirty-first Legislature gave us all these things, but they were cut out by our late "Excellency," who, having failed to grasp the fundamental importance and great economic value of life-saving and health-protection, sacrificed these measures to a narrow view of "economy" and his own political aspirations.

SENATOR WILLACY has introduced in the Legislature a bill to give to the convict's family the net proceeds of his labor. That is a most righteous move, and the wonder is that the world has been so long doing this simple act of justice.

## Figures and Tables

**Figure f1:**